# WIF1 enhanced dentinogenic differentiation in stem cells from apical papilla

**DOI:** 10.1186/s12903-018-0700-6

**Published:** 2019-01-28

**Authors:** Haifeng Wang, Yu Cao

**Affiliations:** 1 Laboratory of Molecular Signaling and Stem Cells Therapy, Beijing Key Laboratory of Tooth Regeneration and Function Reconstruction, Capital Medical University School of Stomatology, No. 4 Tiantanxili, Dongcheng District, Beijing, 100050 China; 2 Department of Stomatology, Beijing Bo’ai hospital, China Rehabilitation Research Center, School of Rehabilitation Capital Medical University, No.10 Jiao Men Bei Lu, Beijing, 100068 China

**Keywords:** WIF1, Dentinogenic differentiation, Stem cells from apical papilla (SCAPs)

## Abstract

**Background:**

Odontogenic mesenchymal stem cells (MSCs) isolated from tooth tissues are a reliable resource that can be utilized for dental tissue regeneration. Exploration of the mechanisms underlying the regulation of their differentiation may be helpful for investigating potential clinical applications. The stem cell niche plays an important role in maintaining cell functioning. Previous studies found that Wnt inhibitory factor 1 (WIF1) is more highly expressed in apical papilla tissues than in stem cells from apical papilla (SCAPs) using microarray analysis. However, the function of WIF1 in SCAPs remains unclear. In the present study, we investigated the function of WIF1 during dentinogenic differentiation in SCAPs.

**Methods:**

A retrovirus containing HA-WIF1 was used to overexpress WIF1 in SCAPs. Using Western blot analysis, we verified the expression of HA-WIF1. Alkaline phosphatase (ALP) activity assays, Alizarin Red staining and quantitative calcium analysis were performed to investigate the in vitro potential for dentinogenic differentiation in SCAPs. The expression of dentinogenesis-associated genes DSPP, DMP1, Runx2 and OSX were assayed using real-time RT-PCR. Transplantation experiments were used to measure dentinogenesis potential in vivo.

**Results:**

The real time RT-PCR results showed that WIF1 was more highly expressed in apical papilla tissues than in SCAPs, and its expression was increased during the process of dentinogenic differentiation. Overexpression of WIF1 enhanced ALP activity and mineralization in vitro, as well as the expression of DSPP, DMP1 and OSX in SCAPs. Moreover, in vivo transplantation experiments revealed that dentinogenesis in SCAPs was enhanced by WIF1 overexpression.

**Conclusion:**

These results suggest that WIF1 may enhance dentinogenic differentiation potential in dental MSCs via its regulation of OSX and identified potential target genes that could be useful for improving dental tissue regeneration.

## Background

Due to technological improvements in tissue engineering, stem cell therapy holds great promise for the advancement of regenerative medicine. Mesenchymal stem cells (MSCs) are considered a good cell source for use in tissue regeneration [1]. From tooth tissues, it is possible to isolate several types of MSCs, including periodontal ligament (PDLSCs), dental pulp (DPSCs), and dental follicular stem cells (DFSCs), as well as stem cells from apical papilla (SCAPs) [1–3]. These MSCs are more intimately associated with dental tissues and can regenerate bone/dentin-like tissues and repair damaged dental tissues [1–5]. However, their potential for use in clinical applications is restricted since the mechanisms underlying dentinogenic differentiation remain unclear.

The stem cell niche supports and maintains the functioning of MSCs. This niche cannot be maintained when MSCs are isolated and cultured in vitro using current methods. An impaired niche will not be beneficial for dental MSC-mediated tooth tissue regeneration [6–8]. Therefore, the identification of the key regulator of the niche that supports dental MSCs will aid in the enhancement of dental tissue regeneration. A previous study examined gene expression profiles in apical papilla tissues, SCAPs and SCAP cell sheets and revealed several candidate key genes within the SCAP niche using microarray and bioinformatic analysis [9]. In their analysis, one canonical Wnt/β-catenin inhibitor, Wnt inhibitory factor 1 (WIF1), was found to be more highly expressed in apical papilla tissues than in SCAPs. Wnt proteins are important regulators of differentiation in stem cells; the Wnt/β-catenin pathway enhances osteogenesis of MSCs and osteoprogenitor cells by upregulating osteoblast-related genes [10]. However, another study found that the Wnt/β-catenin pathway inhibited osteogenesis of PDLSCs [11]. This suggests that the Wnt/β-catenin pathway may play different roles in MSC regulation. WIF1 belongs to a family of secreted modulators of Wnt proteins. Other members of the Wnt modulator family, secreted frizzled-related proteins (sFRPs), play different roles in Wnt signaling depending on the cell subtype and model used [12–16]. In fact, WIF1 also has the effect of modulating Wnt signaling. For example, a previous study found that WIF1 inhibits osteoblastic differentiation in mouse mesenchymal C3H10T1/2 cells [17]. Another study found that WIF1 is highly expressed at cartilage-mesenchyme interfaces in the skeleton during early development and that it blocks the classical Wnt signaling pathway and impairs Wnt3a-mediated inhibition of chondrogenesis in embryonic chick limb-bud cells [18]. However, the function of WIF1 during dentinogenic differentiation in dental MSCs remains unclear.

In the present study, we utilized SCAPs to investigate the functioning of WIF1 during dentinogenic differentiation. We found that WIF1 could enhance the dentinogenic differentiation capacity in SCAPs. These findings provide novel insights into the mechanisms underlying directed differentiation of dental tissue-derived MSCs and have valuable clinical applications.

## Methods

### Cell culture of SCAPs and human embryonic kidney (293 T) cells

All our research involving human stem cells complies with the ISSCR “Guidelines for the Conduct of Human Embryonic Stem Cell Research.” Wisdom teeth were obtained from patients who provided written informed consent according to guidelines approved by the ethical committee (Ethical Committee Agreement, Beijing Stomatological Hospital, Ethics Review No. 2011–02). We disinfected the teeth in 75% ethanol and then washed them with phosphate buffered saline (PBS). The cell culture procedures used, as well as the phenotype, lineage markers and lineage differentiation potentials of SCAPs were identified and described in our previous study [19]. Briefly, apical papilla were gently separated from the apical root and then digested in a solution containing 3 mg/mL collagenase type I (Worthington Biochemical Corp., Lakewood, NJ, USA) and 4 mg/mL dispase (Roche Diagnostics Corp., Indianapolis, IN, USA) for 1 h at 37 °C. A single-cell suspension was produced by passing the solution through a 70 μm strainer (Falcon, BD Biosciences, San Jose, CA, USA). The SCAPs were grown in DMEM alpha modified Eagle’s medium (Invitrogen, Carlsbad, CA, USA) that was supplemented with 15% fetal bovine serum (FBS; Invitrogen), 2 mmol/L glutamine, 100 U/mL penicillin, and 100 μg/mL streptomycin (Invitrogen) at 37 °C in a humidified incubator containing 5% CO2. The culture medium was changed every 3 days.

Human embryonic kidney 293 T cells were maintained in complete DMEM medium containing 10% fetal bovine serum (FBS; Invitrogen), 100 U/mL penicillin, and 100 μg/mL streptomycin (Invitrogen).

### Plasmid construction and viral infection

Full-length human *WNT1* cDNA containing a hemagglutinin (HA) tag was produced using a standard gene synthesis method and subcloned into the pQCXIN retroviral vector (BD Clontech, Mountain View, CA, USA) between the Age I and EcoR1 restriction sites and verified by genetic sequencing. The viral packaging was then performed in 293 T cells according to the manufacturer’s protocol (BD Clontech). Prior to viral infections, the SCAPs were subcultured overnight and then infected with retroviruses in the presence of polybrene (6 μg/ml; Sigma-Aldrich, St. Louis, MO, USA) for 12 h. After 48 h, infected cells were selected using 600 mg/ML G418 (Sigma-Aldrich).

### Reverse transcriptase-polymerase chain reaction (RT-PCR) and real-time RT-PCR

Total RNA was isolated from SCAPs using Trizol reagent (Invitrogen). cDNA was synthesized from a 2 μg aliquot of RNA containing oligo(dT), and reverse transcriptase(Invitrogen) according to the manufacturer’s protocol. Real-time PCRs were performed using the QuantiTect SYBR Green PCR kit (Qiangen, Hilden, Germany) and the Bio-Rad Real-time PCR Detection System. The changes in gene expression were determined using the 2^-△△**CT**^ method. The primers used to specific genes are shown in Table 1.

**Table 1:** Primers sequences used in the Real-time RT-PCR

Gene Symbol	Primer Sequences (5′--3′)
GAPDH-F	CGAACCTCTCTGCTCCTCCTGTTCG
GAPDH-R	CATGGTGTCTGAGCGATGTGG
DSPP-F	TTCCGATGGGAGTCCTAGTG
DSPP-R	TCTTCTTTCCCATGGTCCTG
DMP1-F	GTCCCCTGAGGATGAGAACA
DMP1-R	CCTCGCTCTGACTCTCTGCT
OSX-F	GCCAGAAGCTGTGAAACCTC
OSX-R	AGGGAGATGGGGTACATTCC
WIF1-F	CAAAGCAAACTGCTCAACCA
WIF1-R	CTCCATTTCGACAGGGTTGT

### In vitro cell differentiation assay

Osteo/dentinogenesis differentiation assays were performed as previously reported [4]. The expression of the DSPP, DMP1, OSX and Runx2 genes was determined using real-time RT-PCR.

### Alkaline phosphatase and alizarin red detection

Mineralization was induced in SCAPs using the STEMPRO Osteogenesis Differentiation Kit (Invitrogen). ALP activity was assayed at 5 days using an ALP activity kit according to the manufacturer’s protocol (Sigma-Aldrich). For the mineralization assay, the cells were induced for 2 weeks, fixed in 70% ethanol and then stained with 2% Alizarin red (Sigma-Aldrich). To quantitatively measure calcium, the Alizarin Red-stained cells were then destained with 10% cetylpyridinium chloride in 10 mM sodium phosphate for 30 min at room temperature and measured at 562 nm in a multiplate reader; the amount of calcium was determined via comparison to a standard calcium curve obtained using calcium dilutions prepared in the same solution. The final calcium level in each group was normalized to the total protein concentration measured in a duplicate plate.

### Western blot analysis

The cells were lysed in RIPA buffer (10 mM Tris-HCL, 1 mM EDTA, 1% sodium dodecylsulfate (SDS), 1% NP-40, 1:100 proteinase inhibitor cocktail, 50 mM β-glycerophosphate, 50 mM sodium fluoride). The samples were separated on a 10% SDS polyacrylamide gel and transferred to a polyvinylidene difluoride (PVDF) membrane using a semidry transfer apparatus (Bio-Rad, Hercules, CA,USA). The membranes were blotted with 5% dehydrated milk for 2 h and then incubated with primary antibodies overnight. The membranes then were incubated with horseradish peroxidase-conjugated anti-rabbit or anti-mouse IgG (Promega, Madison, WI, USA) and visualized using SuperSignal reagents (Pierce, Rockford, IL, USA). The primary antibodies used were anti-HA (Clone No. C29F4, Cat No. 3724, Cell Signaling Technology, Beverly, MA, USA) and anti-glyceraldehyde 3-phosphate dehydrogenase (GAPDH; Clone No. GAPDH-71.1, Cat No. G8795, Sigma-Aldrich), which is a housekeeping gene.

### Transplantation in nude mice

These procedures were performed with the approval of the Ethics Committee and Animal Care and Use Committee of Beijing Stomatological Hospital at Capital Medical University (AEEI-2015-085). The animals were purchased from the Institute of Animal Science at Vital River Co., Ltd. and were not treated with drugs or subject to any other procedures prior to transplantation. Approximately 2.0 × 10^6^ cells were mixed with 20 mg of HA/tricalcium phosphate ceramic particles (Engineering Research Center for Biomaterials, Sichuan University, Chengdu, China) and then transplanted subcutaneously beneath the dorsal skin of 10-week-old immunocompromised beige mice (nu/nu nude mice). Eight weeks later, the tissues were harvested, fixed in 10% formalin, decalcified using 10% EDTA buffer (pH 8.0), and embedded in paraffin.

### Immunohistochemical staining

Five μm sections were cut from tissue blocks, deparaffinized and hydrated. The sections were then soaked in 3% H_2_O_2_ for 10 min and washed 3 times in distilled water for 5 min each. Epitope retrieval was conducted for 10 min in a microwave oven using 1.0 mmol/L EDTA buffer (pH 8.0), followed by a 1-h cool down period. The sections were then washed 5 times with phosphate-buffered saline (PBS) for 5 min each time and incubated with 10% goat serum for 60 min to block nonspecific antibody binding. After washing with PBS, the sections were incubated with a primary monoclonal antibody against DSPP (sc-33,586, Santa Cruz) overnight at 4 °C. After washing 3 times in PBS for 5 min each, the sections were incubated with horseradish peroxidase–conjugated anti-rabbit secondary antibody (Promega, Madison, WI, USA) for 30 min at room temperature. The sections were washed 3 times in PBS for 5 min each and then subjected to coloration using a diaminobenzidine (DAB) kit (Cat No. 8059S, Cell Signaling Technology, Inc. Beverly, MA, USA). Finally, the sections were counterstained with hematoxylin, dehydrated in a graded series of alcohol washes and mounted for light microscopy with neutral gum.

### Statistical analyses

All data are presented as the mean ± standard deviation (SD). All statistical calculations were performed using SPSS13.0 statistical software. Student’s t-test was performed to determine the statistical significance. A *P*-value ≤0.05 was considered to denote statistical significance.

## Results

### WIF1 expression was increased during dentinogenic differentiation

**Fig. 1:**
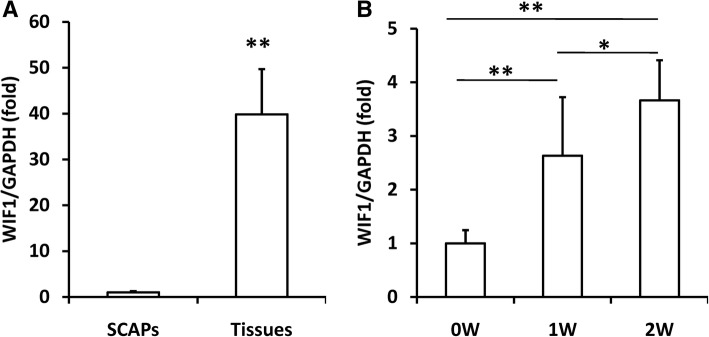
WIF1 expression was increased in apical papilla tissue and in SCAPs during the induction of mineralization. (**a**) Real-time RT-PCR results showing that the expression of WIF1 was much higher in apical papilla tissues than in SCAPs. (**b**) Real-time RT-PCR results showing that the expression of WIF1 was increased during osteo/dentinogenic differentiation in SCAPs. GAPDH was used as an internal control. Student’s t-test was performed to determine the statistical significance. All error bars represent the s.d. (*n* = 3). *P ≤ 0.05; **P ≤ 0.01

We first examined the expression of WIF1 in SCAPs and apical papilla tissues. The real-time RT-PCR results showed that WIF1 expression was much higher in apical papilla tissues than in SCAPs (Fig. 1a). We then measured the expression of WIF1 in differentiated SCAPs upon the induction of mineralization. We found that, when the osteo/dentinogenic differentiation process was induced in SCAPs, WIF1 expression was upregulated during dentinogenic differentiation in a time-dependent manner (Fig. 1b).

### WIF1 enhanced the dentinogenic differentiation potential of SCAPs in vitro

**Fig. 2:**
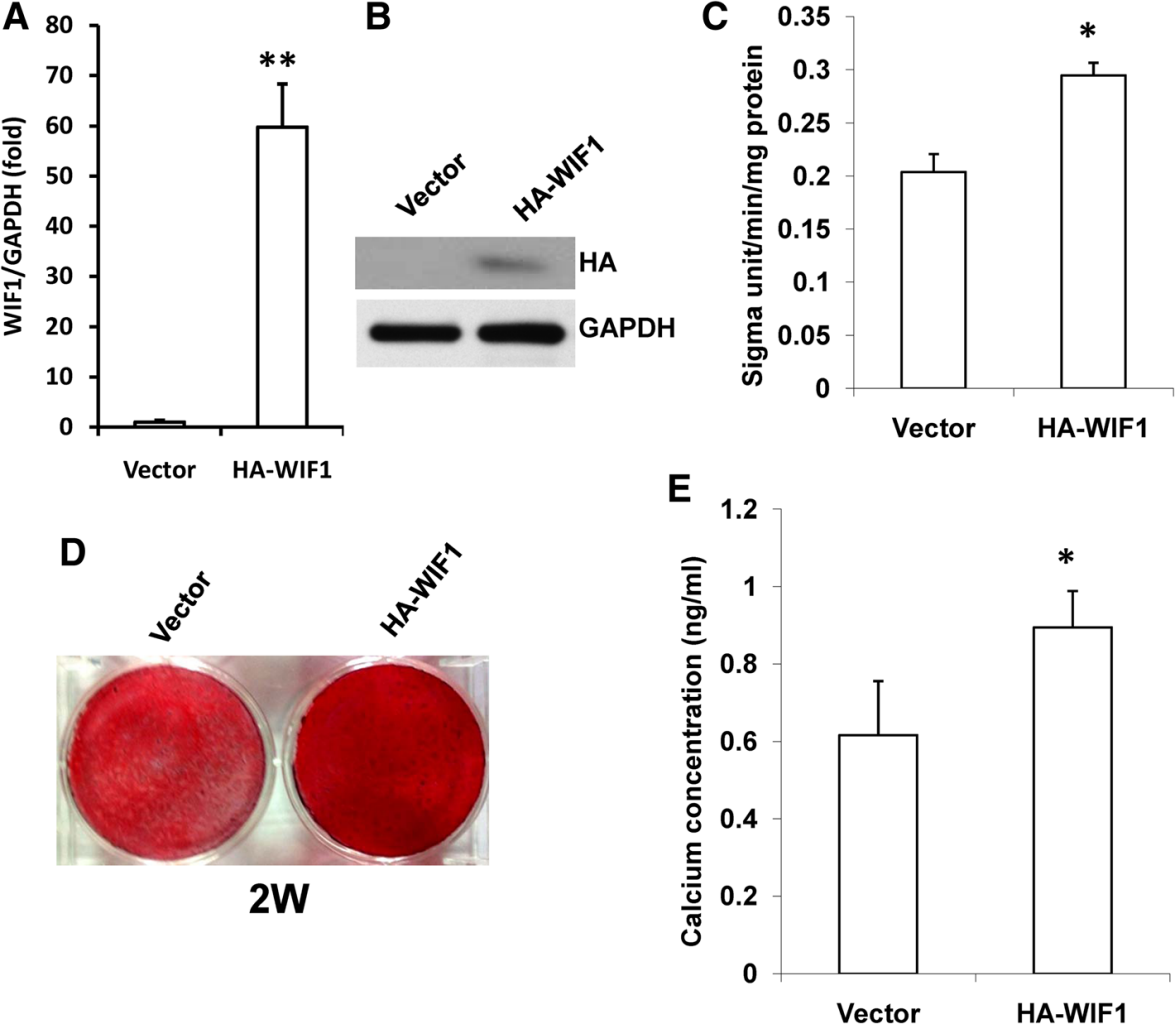
Overexpression of WIF1 enhanced osteo/dentinogenic differentiation in SCAPs. (**a**, **b**) Real-time RT-PCR result (**a**) and Western blot result (**b**) showing that WIF1 was overexpressed in SCAPs due to retrovirus infection. GAPDH was used as an internal control. (**c**) ALP activity assays showing that ALP activity was enhanced in SCAPs that overexpressed WIF1 5 days after mineralization induction. (**d**, **e**) Alizarin Red staining (**d**) and calcium quantitative analysis (**e**) showing that overexpression of WIF1 enhanced mineralization in SCAPs 2 weeks after mineralization induction. Student’s t-test was performed to determine the statistical significance. All error bars represent the s.d. (*n* = 3). *P ≤ 0.05; **P ≤ 0.01

To understand the function of WIF1 during the differentiation of SCAPs, we inserted the WIF1 sequence along with an HA tag into a retroviral vector. This construct resulted in the overexpression of ectopic HA-WIF1 when transduced into SCAPs via retroviral infection. After selection with 600 μg/mL G418 for 14 days, ectopic WIF1 expression was confirmed using real-time RT-PCR and Western blot analysis (Fig. 2a, b). Next, the transduced SCAPs were cultured in mineralization-inducing medium to investigate their dentinogenic differentiation potential. At 5 days after induction, the results indicated that the overexpression of wild-type WIF1 promoted the activity of ALP, which is an early marker of dentinogenic differentiation in SCAPs (Fig. 2c). This was confirmed by the observation that mineralization was significantly enhanced in SCAPs that overexpressed WIF1 after 2 weeks of induction compared to cells infected with the empty vector, as determined using Alizarin Red staining and quantitative calcium measurement (Fig. 2d, e).

**Fig. 3:**
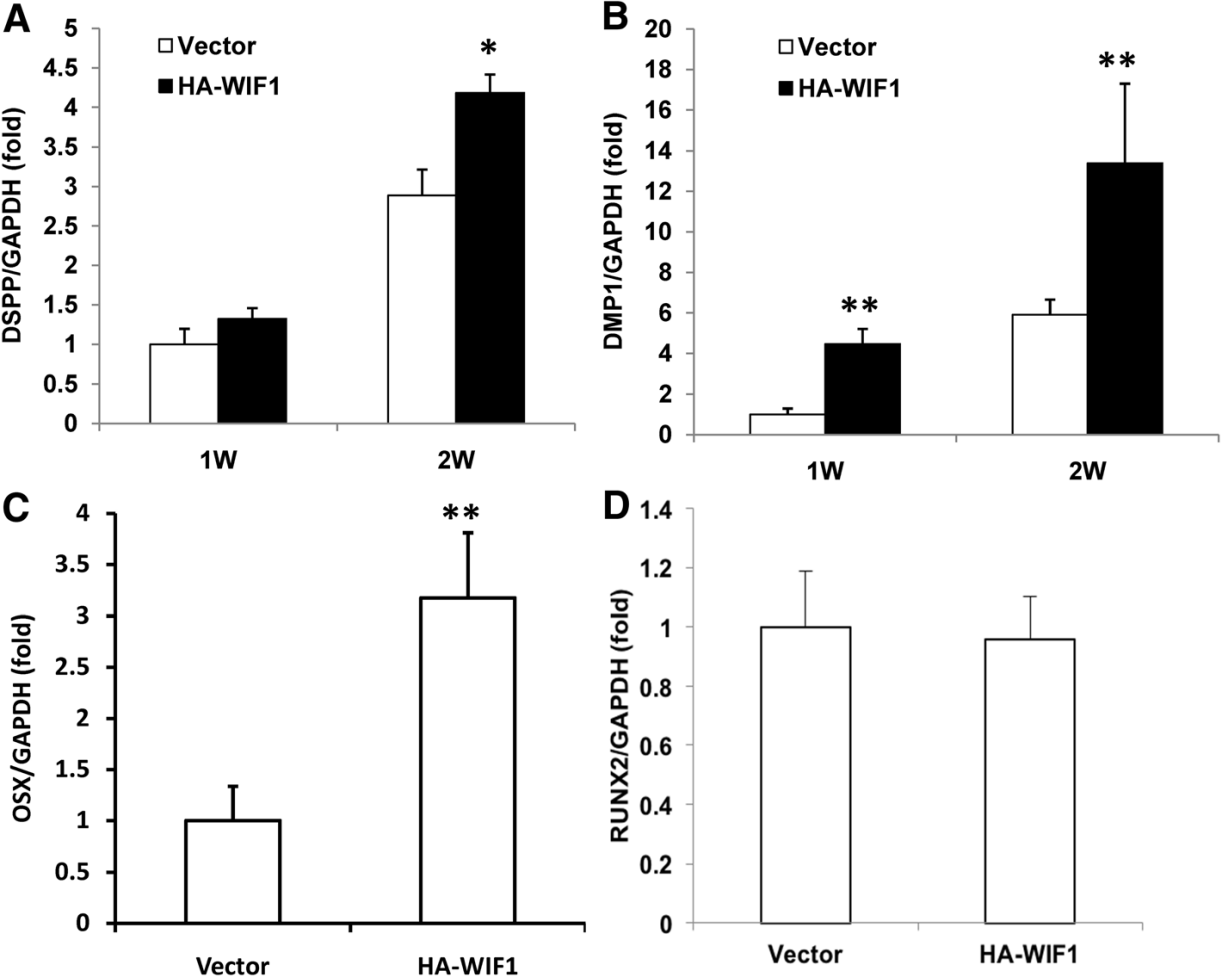
Overexpression of WIF1 increased the expressions of DPM1, DSPP and OSX in SCAPs. Real-time RT-PCR results showing that the expression of DSPP (**a**) DMP1 (**b**), and OSX **(c)** were increased in WIF1 overexpressing SCAPs compared to control SCAPs. **(d)** Real-time RT-PCR results showing that the expression of RUNX2 was not significantly altered by WIF1 overexpression. GAPDH was used as an internal control. Student’s t-test was performed to determine the statistical significance. All error bars represent the s.d. (n = 3). *P ≤ 0.05; **P ≤ 0.01

The dentinogenic marker genes, DSPP and DMP1, were subsequently measured. The real-time RT-PCR results showed that DSPP was induced to a greater extent in SCAP-WIF1 cells than in SCAP-Vector cells after 2 weeks of induction (Fig. 3a), and DMP1 was induced to a greater extent in SCAP-WIF1 cells than in SCAP-Vector cells at 1 and 2 weeks after induction (Fig. 3b). We also examined the expression of key transcription factors involved in dentinogenic differentiation in SCAPs, including runt-related transcription factor 2 (known as RUNX2) and OSX. Our results showed that the mRNA levels of OSX were significantly higher in SCAP-WIF1 cells than in SCAP-Vector cells (Fig. 3c), but that the expression of RUNX2 was not significantly altered (Fig. 3d).

### WIF1 enhanced in vivo dentinogenesis in SCAPs

**Fig. 4:**
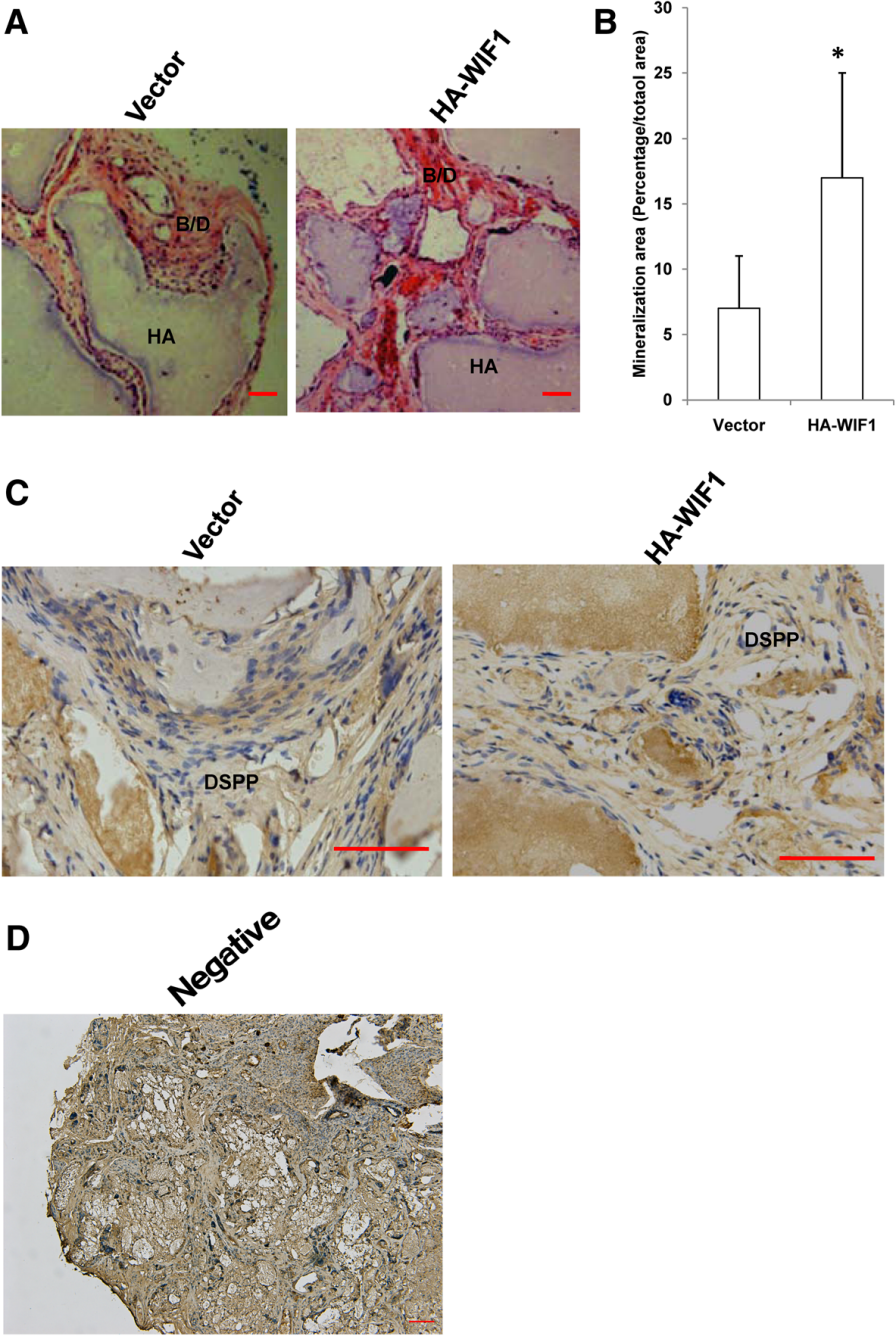
Overexpression of WIF1 enhanced dentinogenesis in SCAPs. SCAPs were transplanted subcutaneously into immunocompromised mice and monitored for 8 weeks. (**a**) H&E-stained micrographs showing bone/dentin-like tissue formation. (**b**) Qualitative measurement of bone/dentin-like tissues in tissue samples. (**c**) Immunohistochemical results demonstrating DSPP expression. (**d**) Immunohistochemical results showing negative control staining in the absence of primary antibody. All error bars represent the s.d. (*n* = 5). Bar: 100 μm. **P ≤ 0.05. B/D, bone/dentin-like tissues; HA, hydroxyapatite tricalcium carrier

We investigated whether the overexpression of WIF1 would affect SCAP dentinogenesis in vivo. We transplanted control or WIF1 SCAPs subcutaneously into nude mice. The transplanted tissues were harvested eight weeks after transplantation. H&E staining revealed that a greater amount of bone/dentin-like tissues were present in the mice transplanted with WIF1 SCAPs than in mice transplanted with control SCAPs (Fig. 4a). Qualitative measurements also revealed a greater amount of bone/dentin-like tissues in mice transplanted with WIF1 SCAPs than in those transplanted with control SCAPs (Fig. 4b). These transplantation experiments demonstrated that WIF1 SCAPs generated a greater amount of bone/dentin-like tissues than control SCAPs in vivo.

Immunohistochemical staining was then used to measure the expression of DSPP. The results revealed that more DSPP protein was present in tissues transplanted with SCAP-WIF1 cells than those transplanted with SCAP-Vector cells (Fig. 4c). Additionally, negative control staining was shown to occur in the absence of primary antibody (Fig. 4d). Overall together, these results showed that the overexpression of WIF1 significantly enhanced the formation of dentin-like tissues.

## Discussion

MSCs derived from dental tissues have proved to be promising source of cells with therapeutic potential, especially for use in dental tissue treatment. However, little is known about the molecular mechanisms underlying the stability and differentiation potential of dental MSCs. SCAPs are important for tooth development and regeneration. A key factor that ensures successful dental tissue regeneration is the niche in which the dental cells and tissues are grown [20], which makes it important to identify the key genes that support the functioning of MSCs in the niche. The commitment and differentiation of MSCs is reliant upon Wnt/β-catenin signaling [21–24]. Therefore, we chose to investigate the Wnt/β-catenin modulator WIF1, which has been found to be more highly expressed in apical papilla tissues than in SCAPs using microarray analysis. First, we confirmed that WIF1 expression was decreased in cultured SCAPs after isolation from apical papilla and then verified that it was increased during the dentinogenic differentiation process; this indicated that increased levels of WIF1 may play a role in dentinogenic differentiation.

Based on these observations, we investigated the function of WIF1 during the dentinogenic differentiation process in SCAPs. The results showed that WIF1 promoted ALP activity and mineralization in vitro*.* ALP is as an indicator of early differentiation during the osteo/dentinogenic process [25]. The presence of the mineralization phenotype is an indicator of the end stage of the osteo/dentinogenic differentiation process. Moreover, transplantation experiments demonstrated that newly formed bone/dentin-like tissues were deposited by transplanted SCAPs-Vector and SCAPs-WIF1 cells and revealed that WIF1 promoted osteo/dentinogenesis in vivo. These results indicated that WIF1 enhanced osteo/dentinogenic differentiation in SCAPs. To clarify the role of WIF1 in dentinogenic differentiation, we also investigated dentinogenic differentiation indicators. DSPP and DMP1 are classic odontogenic markers; DSPP is a key gene involved in the process of dentin formation, while DMP1 has been shown to regulate DSPP [26–28]. We found that the expression of DSPP and DMP1 were enhanced by WIF1 in SCAPs in vitro. Additionally, a greater amount of DSPP protein was found in tissues, transplanted with SCAPs-WIF1 cells. These results indicated that WIF1 was able to promote dentinogenic differentiation in SCAPs.

In addition, we found that expression of the transcription factor OSX was also enhanced by WIF1. OSX is known to be an essential transcription factor that contains three C2H2-type zinc finger DNA binding domains. Osx is expressed during the entire process of tooth development [29–31]. The amount of cementum has been found to be reduced due to Osx deletion in mice [32]. An in vitro study found that Osx increases Dspp transcription in odontoblast-like cells [33]. This evidence suggests that Osx plays a critical role in dentinogenic differentiation and formation. We also found that the mRNA expression level of RUNX2, a transcription factor, was not significantly different in SCAP-WIF1 and SCAP-Vector cells. An in vitro study by Han found that Wnt/β-catenin could enhance dentinogenic differentiation in DPSC cells by activating RUNX2 [34]. There are no reports suggesting that RUNX2 upregulation is not required for dentinogenic differentiation. Overall, these findings suggested that WIF1 may enhance dentinogenic differentiation via enhancement of OSX expression in SCAPs.

## Conclusion

Our results showed that WIF1 enhanced dentinogenic differentiation in SCAPs by activating the transcription factor OSX. Our work explored the mechanisms underlying the effects of WIF1 on directed differentiation in dental MSCs and provided potential target genes that could be useful in improving dental tissue regeneration using dental tissue-derived MSCs.

## Abbreviations

ALP: Alkaline phosphatase
DFSCs: Stem cells from dental follicles
DMP1: Dentin matrix protein 1
DPSCs: Dental pulp stem cells
DSPP: Dentin sialophosphoprotein
HA: Hemagglutinin
MSCs: Mesenchymal stem cells
OSX: Osterix
PBS: Phosphate-buffered saline
PDLSCs: Periodontal ligament
RT-PCR: Reverse transcriptase-polymerase chain reaction
SCAPs: Stem cells from apical papilla
sFRPs: Secreted frizzled-related proteins
SPSS: Statistical Package for Social Science
WIF1: Wnt inhibitory factor 1
